# Asociación del consumo de cigarro, derivados nicotínicos y cannabis con enfermedad periodontal. Una revisión

**DOI:** 10.21142/2523-2754-1303-2025-255

**Published:** 2025-08-31

**Authors:** Olenka Yomira Valenzuela-Torres, Mauricio André Zapata-Sifuentes, Angela Quispe-Salcedo

**Affiliations:** 1 División de Odontología Preventiva, Departamento de Ciencias de la Salud Bucal, Escuela de Posgrado de Ciencias Médicas y Dentales de la Universidad de Niigata, Niigata, Japón. olenka_v@dent.niigata-u.ac.jp Niigata Japón olenka_v@dent.niigata-u.ac.jp; 2 Escuela de Posgrado de la Facultad de Ciencias de la Salud, Universidad Científica del Sur, Lima, Perú. Lima Perú; 3 Carrera de Estomatología, Universidad Científica del Sur, Lima, Perú. mzapata@dent.niigata-u.ac.jp Lima Perú mzapata@dent.niigata-u.ac.jp; 4 División de Anatomía y Biología Celular de los Tejidos Duros, Departamento de Regeneración y Reconstrucción de Tejidos, Escuela de Posgrado de Ciencias Médicas y Odontológicas de la Universidad de Niigata, Niigata, Japón. aquispesa@dent.niigata-u.ac.jp Niigata Japón aquispesa@dent.niigata-u.ac.jp

**Keywords:** cannabis, cigarrillo, enfermedad periodontal, sistemas electrónicos de liberación de nicotina, Cannabis, cigarette smoking, periodontal disease, electronic nicotine delivery systems

## Abstract

**Antecedentes:**

El tabaquismo es considerado uno de los mayores factores de riesgo de muerte en el mundo. La evidencia científica asegura que fumar está asociado con enfermedades graves y crónicas, como las enfermedades inflamatorias; los cánceres de cabeza, cuello y pulmón; y enfermedades orales, entre las cuales destaca la enfermedad periodontal. El objetivo de esta revisión fue evaluar la asociación entre el consumo de cigarrillos, derivados nicotínicos y cannabis con la enfermedad periodontal.

**Materiales y métodos:**

Se realizo una revisión narrativa de estudios pertenecientes a las bases de datos Medline, Scopus y SciELO; incluyéndose aquellos artículos que incorporaban población adulta.

**Resultados:**

El *screening* y síntesis de la información obtenida de los estudios seleccionados fue llevado a cabo por dos revisores que trabajaron de forma independiente. Se tamizó 519 artículos, de los cuales se incluyeron finalmente 39, que consistieron en estudios transversales, ensayos clínicos aleatorios, mendeliano aleatorizado, cohortes, casos-controles, longitudinales e *in vitro*.

**Conclusión:**

El presente estudio concluye que existe asociación entre la enfermedad periodontal y el consumo de cigarrillos, derivados nicotínicos y cannabis, según la evidencia recolectada. Se sugiere a futuras investigaciones poder evaluar y poner más enfoque a la asociación del cannabis con la enfermedad periodontal, ya que la evidencia es poca a diferencia de otras drogas con la enfermedad periodontal.

## Abstract (en)

**Background:**

Tobacco use is considered one of the leading risk factors for death worldwide. Scientific evidence shows that smoking is associated with serious and chronic diseases, such as inflammatory diseases, head, neck and lung cancers, as well as oral diseases, including periodontal disease. This study aimed to evaluate the association between tobacco, nicotine derivatives, cannabis consumption and periodontal disease.

**Materials and Methods:**

A narrative review was performed based on studies collected in the Medline, Scopus and SciELO databases, considering those studies involving the adult population.

**Results:**

screening and synthesis of the available information was performed by two reviewers working independently. Five hundred and nineteen articles were reviewed, while 39 were finally selected; including cross-sectional studies, randomized clinical trials, mendelian randomization studies, cohort studies, case-control studies, longitudinal studies, and *in vitro* studies.

**Conclusion:**

The current study concluded that there is an association between periodontal disease and the consumption of tobacco, nicotine derivatives, and cannabis based on the available evidence. Future studies should evaluate and place major emphasis on the association of cannabis with periodontal disease, as the evidence is limited compared to other type of drugs.

## INTRODUCCIÓN

El tabaquismo es considerado como uno de los mayores factores de riesgo de muerte en el mundo, aun cuando es también considerada la causa de mortalidad más evitable existente en la actualidad ^1^^-^^3^. En 2021, se estimó que el consumo de tabaco era responsable del 15% de las muertes anuales a nivel mundial ^4^, lo que coincide con la evidencia científica que asocia este hábito con enfermedades graves y crónicas, tales como enfermedades inflamatorias; cánceres de cabeza, cuello y pulmón; y enfermedades orales ^1^^,^^4^^-^^9^. Asimismo, se sabe que el humo del cigarro contiene más de 4000 sustancias químicas, 69 de ellas carcinógenas. Más aún, los riesgos para la salud no se restringen únicamente al fumador, sino también a los individuos expuestos involuntariamente al humo, también llamados fumadores pasivos ^4^^,^^10^^,^^11^. También se ha asociado a problemas cardiovasculares, respiratorios y cerebrovasculares ^1^^,^^12^^-^^15^.

El inicio del contacto con el tabaco suele tener lugar en la adolescencia debido a la presión social y la copiosa publicidad en redes sociales. Posteriormente, gran parte de los consumidores adultos se vuelven dependientes a consumir tabaco por la presencia de la nicotina, la cual es adictiva ^4^. Debido al impacto del tabaquismo en la salud, se han tomado acciones intensivas para disminuir su prevalencia, tales como programas e incentivos para dejar el tabaquismo, el aumento del precio e impuestos de los productos ^1^^-^^4^. En la última década, nuevos productos emergieron como alternativa a los cigarrillos convencionales. Aunque el concepto fue patentado en los años 60, la introducción al mercado de estos derivados nicotínicos se dio a partir de 2006 ^4^^,^^16^^-^^19^. En la actualidad existe una gran variedad de productos nicotínicos, tales como chicles, pastillas, parches y sistemas electrónicos de administración de nicotina (SEAD), los cuales se han vuelto populares entre los fumadores de todas las edades, y se expandieron a lo largo del mundo desde 2014, al venderse como una alternativa más segura a la combustión del cigarrillo convencional ^5^^,^^6^^,^^9^^,^^16^^,^^18^^-^^24^. 

Por otra parte, el cannabis es la segunda sustancia más consumida después del tabaco y la droga más consumida a nivel mundial. Su presentación se da principalmente en la forma de marihuana, la cual es una mezcla de color verde de flores secas de *Cannabis sativa*^25^^-^^28^. El cannabis ejerce su efecto a través de la interacción con receptores específicos CB1, los cuales se encuentran principalmente en la corteza del cerebro, áreas límbicas, cerebelo y áreas talámicas, y modulan la actividad neuronal. De este modo, es capaz de alterar los receptores que regulan el placer, la memoria, concentración y la percepción sensorial ^27^. Los receptores CB2 también están vinculados al cannabis, y se encuentran en células del sistema inmune, como los macrófagos ^27^. Además, un estudio de 2015 reportó la existencia de la asociación del consumo de cannabis en combinación con tabaco, ya que el 90% de los consumidores de cannabis también fuman tabaco ^25^^,^^27^^,^^29^. Del mismo modo, existe evidencia sólida de una asociación entre el consumo de cannabis con la enfermedad periodontal. así como lesiones reportadas debido a la temperatura del humo, que es incluso mayor que la de los cigarrillos ^25^^,^^29^^,^^30^. Otros efectos secundarios son la xerostomía, las infecciones por *Candida albicans*, estomatitis por nicotina, leucoedema oral y cáncer oral ^27^.

La enfermedad periodontal se define como una afección inflamatoria, crónica y multifactorial, provocada generalmente por la acumulación de biofilm ^10^ que afecta las estructuras de soporte de los dientes (encías, ligamento periodontal y el hueso alveolar), y es la manifestación patológica una respuesta del huésped contra la invasión bacteriana ^6^^,^^8^^,^^9^^,^^14^^,^^21^^,^^22^^,^^27^^,^^31^^-^^35^. La enfermedad periodontal causa una inflamación crónica de los tejidos gingivales, la cual puede progresar hasta degradar el tejido conectivo y hueso en el que se sostienen los dientes, y existe la posibilidad de la pérdida dental ^32^^,^^33^^,^^35^. La prevalencia de esta enfermedad se mantiene sin cambios desde los años 90 ^8^, pues afecta aproximadamente al 50% de la población adulta, incluyendo un 10% de individuos con diagnóstico de periodontitis severa ^25^. Por ejemplo, en Latinoamérica, la periodontitis afecta hasta al 62,6% de las personas en edad adulta, mientras que el 35% de la población infantil presenta gingivitis ^29^. 

La progresión de la enfermedad periodontal está asociada con múltiples patologías sistémicas, tales como enfermedades cardiovasculares, renales crónicas, pulmonares, diabetes, obesidad, parto prematuro y la enfermedad de Alzheimer ^9^^,^^16^^,^^32^^-^^35^. Aunque la principal causa sea la acumulación de biofilm, existen diversos factores de riesgo para el desarrollo y progresión de la enfermedad periodontal ^6^. Entre los principales se encuentra el tabaquismo, la diabetes y la obesidad ^10^^,^^22^^,^^25^^,^^27^; en tanto que el sistema inmune, la condición genética, el estatus socioeconómico, el sexo y la edad son considerados factores predisponentes ^33^^,^^34^. En estos factores, el tabaquismo se considera como el más prevalente, pero evitable ^10^.

A lo largo de los años, numerosas revisiones narrativas y sistemáticas que describen la asociación entre la enfermedad periodontal y el consumo de cigarrillos convencionales y electrónicos, así como los derivados nicotínicos, han sido publicadas en revistas científicas dentro y fuera del campo odontológico. Del mismo modo, y aunque en menor número, se han realizado revisiones acerca del efecto del consumo de cannabis en la salud oral. No obstante, hasta la fecha solo existe una revisión, realizada en 2021, que engloba los efectos de los cigarrillos convencionales, electrónicos, derivados nicotínicos y cannabis sobre la enfermedad periodontal ^4^. Al tratarse de un tema muy relevante, debido a su alarmante epidemiología, el objetivo de este estudio fue proveer información actualizada y conjunta respecto de la asociación entre el consumo de cigarrillos, derivados nicotínicos y cannabis con la enfermedad periodontal, a través de una revisión narrativa.

## MATERIALES Y MÉTODO

### Diseño de estudio

El presente estudio representa una revisión narrativa para realizar un análisis y síntesis de los resultados científicos encontrados hasta el momento, en relación con la asociación del consumo de cigarro, los derivados nicotínicos y el cannabis con la enfermedad periodontal. Este estudio se basó en la metodología PRISMA.

### Criterios de elegibilidad

Se incluyeron aquellos estudios cuya población representaba adultos, de 18 años a más. Asimismo, las investigaciones incluidas debían estar relacionadas con el tema a abordar, es decir, la asociación del consumo de cigarros, derivados nicotínicos y cannabis con la enfermedad periodontal. No hubo limitación en cuanto al país, por lo que la población podría pertenecer a cualquier parte del mundo. Los estudios debían contar como mínimo con algún derivado nicotínico o consumo de cannabis, y definir la asociación de estos con la enfermedad periodontal. Se excluyeron aquellos que estaban basados en revisiones narrativas, revisiones sistemáticas y metaanálisis, así como las cartas al editor.

### Fuentes de información

Se llevó a cabo una búsqueda bibliográfica en diferentes bases de datos como Medline, Scopus y SciELO. Además, se hizo una revisión en cada referencia bibliográfica de los estudios hallados para incluir aquellos con información fundamental en la presente revisión.

### Estrategia de búsqueda

La búsqueda fue llevada a cabo a partir del 18 de octubre de 2023. Hubo restricciones en cuanto al idioma, siendo el inglés y el español los idiomas considerados debido al dominio de estos por los revisores (OV y MZ). Y en cuanto a las fechas, se consideró el periodo de publicación más actual referente al tema, que osciló entre los años 2013 y 2024. Como palabras clave de búsqueda en español se seleccionaron aquellos equivalentes a los términos de búsqueda en el idioma inglés: “Cigarette”; “Electronic Nicotine Delivery Systems”; “Cannabis”; y “Periodontal disease” y se usó como operadores booleanos OR y AND. A continuación, se detallan las estrategias de búsqueda en las bases de datos seleccionadas: En Medline: (((Cigarette[MeSH Terms]) OR (Electronic Nicotine Delivery Systems[MeSH Terms])) OR (Cannabis[MeSH Terms])) AND (periodontal diseases[MeSH Terms]); en SciELO: (nicotine OR Cannabis) AND (periodontal diseases), y en Scopus: (TITLE-ABS-KEY (nicotine) OR TITLE-ABS-KEY (cannabis) AND TITLE-ABS-KEY (periodontitis)) (Tabla 1).

**Tabla 1:** Estrategia de búsqueda

Medline (18/10/2023) n = 213
(((Cigarette[MeSH Terms]) OR (Electronic Nicotine Delivery Systems[MeSH Terms])) OR (Cannabis[MeSH Terms])) AND (periodontal diseases[MeSH Terms])
SciELO (18/10/2023) n = 2
(nicotine OR Cannabis) AND (periodontal diseases)
Scopus (18/10/2023) n = 324
(TITLE-ABS-KEY (nicotine) OR TITLE-ABS-KEY (cannabis) AND TITLE-ABS-KEY (periodontitis)

### Selección de estudios

Al concluir la búsqueda de artículos, estos fueron descargados de cada base de datos y se procedió a importarlos a EndNote Basic (versión web) para la eliminación de duplicados. Posteriormente, se realizó el primer *screening* analizando el título y resumen de los estudios, tarea que fue realizada por dos revisores (OV y MZ) que trabajaron de forma independiente. Luego, se procedió al segundo *screening*, el cual involucró el análisis del contenido, excluyendo revisiones narrativas, revisiones sistemáticas y cartas al editor. También se evaluó si los estudios no cumplían con los criterios de inclusión anteriormente mencionados. Para estas etapas de tamizaje, se empleó el programa Microsoft Excel, en el que, de forma manual, se evaluó de forma detallada cada contenido de los estudios por analizar.

Debido a que el resultado final de la búsqueda realizada podría considerar más de un estudio en cada sección de la presente investigación, se optó por extraer los resultados más relevantes por artículo. Esto fue desarrollado por los mismos revisores que hicieron el primer y segundo *screening* de forma independiente, para luego ser estos discutidos en la selección de los estudios.

### Extracción de información

Toda la información importante fue extraída usando una ficha digital elaborada en Microsoft Excel por los autores del presente estudio. En esta ficha se incluyeron los datos de nombre del autor, título, año de publicación, revista científica, tipo de estudio, tipo de droga utilizada por la población en el estudio, conclusión y recomendaciones para futuros estudios. Asimismo, se clasificaron los estudios según el año de antigüedad desde el más actual al más antiguo. Esta tarea se llevó a cabo de forma duplicada por dos autores (OV y MZ), y se extrajo la información de cada investigación para su verificación y contrastación.

## RESULTADOS

**Figura 1:**
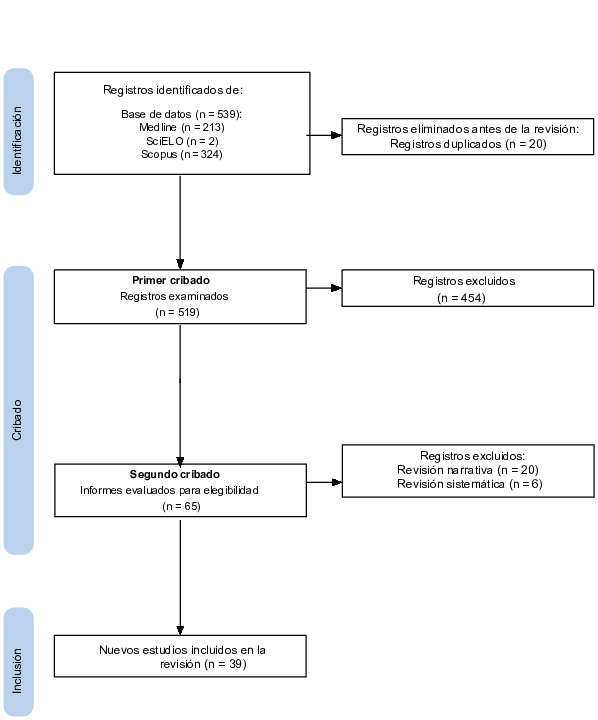
Flujograma de la selección de artículos incluidos en el presente estudio

Durante la selección de estudios, se encontraron 539 artículos en la búsqueda inicial en Medline (n = 213), Scopus (n = 324) y SciELO (n = 2), eliminándose 20 duplicados y resultando 519 artículos. Durante el primer *screening*, se incluyeron sólo 65 artículos donde se llevó a cabo la extracción y se escogió aquellos estudios más relevantes y acorde a los criterios anteriormente señalados. Finalmente, fueron seleccionados 39 artículos que involucraron estudios transversales (n = 17), estudios de ensayos clínicos aleatorios (n = 11), estudio mendeliano aleatorizado (n = 1), estudios de cohortes (n = 3), estudios de caso-control (n = 2), estudios longitudinales (n = 2) y estudios *in vitro* (n = 3) publicados entre los años 2013 y 2024. Los detalles de los estudios excluidos fueron representados a través de un flujograma ilustrado en la Figura 1. Además, la clasificación de los estudios incluidos según las características presentes se muestra en la Tabla 2. 

**Tabla 2:** Características de los estudios incluidos

Autor y año	País	Tipo de estudio	Año de recolección	Tipo de droga	Tamaño muestral
Nasir *et al*. (2023)	Pakistán	Estudio transversal	2019	Tabaco	377
Park *et al*. (2023)	EE. UU.	Estudio transversal	2017-2018	Cigarrillo electrónico	150
Ahmed *et al*. (2022)	Arabia Saudita	Ensayo de control aleatorio	2021	Cigarrillo	60
Alhumaidan *et al*. (2022)	Arabia Saudita	Ensayo de control aleatorio	2021	Cigarrillo	48
Ali *et al.* (2022)	EE. UU.	Ensayo de control aleatorio	2022	Cigarrillo electrónico	75
Alqahtani *et al*. (2022)	Arabia Saudita	Estudio transversal	2021	Cigarrillo y cigarrillo electrónico	150
AlQobaly *et al*. (2022)	Arabia Saudita	Estudio transversal	2015-2018	Cigarrillo electrónico	8129
Basudan *et al*. (2022)	India	Ensayo de control aleatorio	2022	Cigarrillo	68
Thomas *et al*. (2022)	EE. UU.	Cohortes	2022	Cigarrillo electrónico	84

### Cigarrillo convencional y enfermedad periodontal

Se han descrito en numerosos ensayos aleatorios los diversos efectos nocivos del tabaquismo sobre la salud sistémica y oral. El tabaquismo es considerado un factor predisponente y agravante de la enfermedad periodontal y otras patologías más severas, como el cáncer oral ^1^^-^^3^^,^^7^^,^^12^^,^^20^^,^^36^^-^^38^. En Estados Unidos, la enfermedad periodontal tiene una prevalencia del 40% y, en fumadores, se presenta con un porcentaje 50% mayor al de no fumadores ^39^. Fumar cigarrillos favorece el desarrollo de bacterias patógenas que son características de la enfermedad periodontal ^2^^,^^9^. Se ha reportado que fumadores crónicos tienen una mayor pérdida de tejido conectivo y hueso alveolar, así como un número mayor de bolsas periodontales y presencia aumentada de cálculo gingival ^3^^,^^6^. Son diversos los mecanismos propuestos por los cuales el tabaquismo afecta la salud periodontal. Se incluyen la respuesta inmunológica, la composición de la microbiota y la capacidad de reparación tisular del periodonto ^3^. En pacientes fumadores, se ha reportado una modificación en la composición del biofilm subgingival con un incremento de patógenos periodontales, tales como P*orphyromonas gingivalis*, *Tannerella forsythia*, *Treponema denticola*, *Aggregatibacter actinomycetemcomitans*, *Fusobacterium nucleatum* y *Prevotella intermedia*^3^^,^^20^^,^^39^.

El sangrado al sondaje es reducido debido a una alteración del flujo sanguíneo periférico causado por la nicotina y el humo del cigarrillo ^3^^,^^6^^,^^38^^,^^40^. La exposición de los tejidos periodontales al humo del tabaco, el cual contiene más de 4000 químicos (entre estos, 69 carcinógenos) afecta la homeostasis ósea, la reparación de los tejidos y la respuesta inmune, lo que provoca vasoconstricción periférica y fibrosis gingival ^3^^,^^10^^,^^38^. Un estudio histopatológico de 2021 confirmó el engrosamiento del epitelio con hiperqueratosis, edema intraepitelial, necrosis celular intraepitelial y la numerosa presencia de linfocitos T y B en el tejido conectivo ^39^. El humo del cigarrillo puede obstaculizar los mecanismos de estrés oxidativo del periodonto, lo cual inhibe la defensa contra la placa bacteriana y retarda la cicatrización ^39^.

Por otra parte, la nicotina puede alterar la migración y proliferación de fibroblastos gingivales, así como la expresión de moléculas de adhesión de neutrófilos. Tiene la capacidad de acelerar la destrucción del hueso alveolar y retrasar su reparación al inhibir la diferenciación de las células del ligamento periodontal, mediante la inhibición de la producción de colágeno tipo 1 y fibronectina ^10^^,^^37^^-^^40^. Además, es importante destacar que el componente adictivo de los cigarrillos es la nicotina, cuya abstinencia provoca síntomas como ansiedad, estrés y desequilibrio metabólico ^5^. A esto se suman algunos factores que pueden agravar el efecto del tabaquismo en la enfermedad periodontal, como la mala higiene, la edad, la progresión de la enfermedad, la duración y frecuencia del consumo de cigarrillos, y la exposición directa o indirecta ^6^^,^^33^.

### Derivados nicotínicos (ENDS) y enfermedad periodontal

Si bien se considera que los derivados nicotínicos y los cigarrillos electrónicos pueden ser una alternativa viable y “menos dañina” que los cigarrillos convencionales, el consumo prolongado de estos dispositivos está asociado con la enfermedad periodontal, así como con enfermedades respiratorias, cardiovasculares y con cáncer de pulmón y cabeza y cuello en menor medida ^1^^,^^5^^,^^6^^,^^9^^,^^20^^,^^41^. Se ha reportado que el índice de placa, la profundidad de sondaje y la pérdida de hueso alveolar son mayores en pacientes fumadores de ENDS ^5^. Con una amplia diferencia, los diversos sistemas de administración de nicotina, así como los cigarrillos electrónicos, son preferidos por una población joven, en muchos casos adolescente. Esto, junto a otros factores, tales como los indicadores socioeconómicos, la diabetes y la higiene dental, permiten el progreso de la enfermedad periodontal ^1^. Los derivados nicotínicos incluyen una amplia variedad de productos y dispositivos disponibles en el mercado, entre ellos cigarrillos electrónicos ^20^, shisha ^35^, tabaco masticable, ^42^ entre otros.

De estos ejemplares, los cigarrillos electrónicos han ganado popularidad en los últimos años entre la población joven debido a su presentación similar a la de un USB o bolígrafo ^2^. Son dispositivos en los que un tanque lleno de líquido (que contiene nicotina, aminas aromáticas, aldehídos, propilenglicol, glicerina, etanol, acetol y óxido de propileno) está conectado a una batería ^1^^,^^2^^,^^6^^,^^20^^,^^36^^,^^43^, la cual genera una corriente eléctrica que, al calentar el líquido, produce un vapor o aerosol que puede ser inhalado ^2^^,^^20^^,^^36^^,^^43^. Los componentes, al ser liberados en el vapor o aerosol, se adhieren a las estructuras y epitelio de la cavidad oral, nasal y pulmones ^2^^,^^35^^,^^43^. Así, el consumo de cigarrillos electrónicos produce lesiones y alteraciones en el ligamento periodontal, el tejido gingival y los fibroblastos ^6^.

La nicotina contenida y liberada por los cigarrillos electrónicos inhibe la proliferación celular, lo que afecta a los fibroblastos y su diferenciación en miofibroblastos, y con ello retrasa la cicatrización de heridas ^6^^,^^9^^,^^31^. Provoca disbiosis, al aumentar las UFC de *Porphyromonas gingivalis* y *Aggregatibacter actinomycetemcomitans* en el biofilm oral, lo cual origina la inflamación gingival ^9^^,^^20^. El componente adictivo también es destacable, pues empieza como una alternativa para abandonar el tabaquismo convencional, pero termina convirtiéndose en una adicción peligrosa a los cigarrillos electrónicos ^20^. Los aerosoles y saborizantes emitidos por estos dispositivos pueden aumentar el estrés oxidativo en los tejidos periodontales debido a los productos finales de la glicación avanzada ^5^^,^^6^. La temperatura alta del cigarrillo electrónico y sus vapores son nocivos para el epitelio gingival y los labios, y que promueven la apoptosis celular, la necrosis y el daño crónico del ADN en el epitelio ^2^^,^^6^^,^^31^. Se ha reportado una elevación de los niveles de citoquinas salivales, tales como la interleuquina IL-6 y la IL-1β en consumidores de cigarrillos electrónicos ^2^^,^^6^^,^^20^, así como otros marcadores de inflamación, por ejemplo, el receptor de ligando B NF-kappa, factor de necrosis tumoral alfa, tanto en saliva como en el líquido crevicular ^5^. Del mismo modo, se demostró un aumento del nivel de prostaglandinas y metaloproteasas relacionadas con el daño periodontal ^6^.

Entre otros componentes del líquido dentro del dispositivo que son reportados como nocivos se puede mencionar la emisión de aldehídos carbonílicos que provocan la degradación de la matriz periodontal a través de la activación del sistema autoinmune, lo que genera la destrucción del hueso alveolar ^9^. Una alta concentración de nitrosaminas asociadas al tabaco puede dañar irreversiblemente el epitelio gingival que está expuesto ^36^. El saborizante también es nocivo puesto que disminuye la proliferación de fibroblastos en el ligamento periodontal ^31^^,^^44^.

Otros productos con los que se ha reportado una asociación con la enfermedad periodontal incluyen el tabaco masticable, el tabaco en polvo, etc. Sus efectos adversos iniciales son alteración del gusto; decoloración y manchas en los dientes, lengua y mucosa; caries y formación de bolsas periodontales. Masticar tabaco provoca recesión gingival, halitosis y úlceras en la mucosa oral. Usuarios de betel, gutka, naswar mostraron una mayor prevalencia de movilidad dental de moderada a severa, e incluso pérdida de piezas dentarias ^41^.

### Cannabis

En la actualidad, el cannabis es una de las drogas más consumidas después del tabaco y esto se debe a que existen más mercados que tienen la autorización para venderlo. Esta sustancia posee similares componentes químicos a los del tabaco, pero la diferencia radica en que contiene nicotina y cannabinoides ^45^. La semejanza en la constitución del tabaco y el cannabis puede ser un factor de riesgo de la enfermedad periodontal, pues, así como el tabaco, afecta la cavidad bucal, incluido el periodonto.

Son pocas las investigaciones que han evaluado la relación del cannabis con la enfermedad periodontal. En los estudios observacionales, se ha afirmado que el cannabis puede influir en el desarrollo de la periodontitis. El cannabis ha demostrado una asociación con un incremento de sensación de somnolencia y letargo, así como predilección por el dulce, lo que generando una conducta no saludable en la dieta, la higiene bucal y la visita regular al odontólogo; esto implica un mayor riesgo de enfermedad periodontal ^46^. Cabe recalcar que el mecanismo biológico del cannabis genera disminución del flujo salival, alteración del microbioma oral, respuesta inmune deteriorada e incremento de la producción de enzimas y de citocinas destructivas ^47^.

En ensayos clínicos, el cannabis ha demostrado generar malas respuestas inmunes inflamatorias salivales (niveles salivales totales de IL-17A e IL-23), así como en los fumadores de cigarrillos, en comparación con personas no fumadoras sin o con periodontitis ^26^. En estudios experimentales previos, se ha evidenciado pérdida de hueso alveolar y la disminución de la densidad ósea al aspirar marihuana ^48^. Además, se ha notado alto riesgo de bruxismo y lesiones en la cavidad bucal en pacientes fumadores de marihuana ^49^.

Mayormente el cannabis es usado junto al tabaco envuelto en papel fino, con el nombre de “porro”, por lo que no se puede saber con certeza si existe una asociación con el desarrollo de la periodontitis. Sin embargo, se han evidenciado mayores efectos nocivos a nivel molecular al fumar marihuana como “porro” que al fumar simplemente cigarrillos ^26^. Ante ello, los estudios mendelianos aleatorizados sobre este tema evaden los factores de confusión entre la marihuana y el tabaco aprovechando la información genética de la exposición. Dichos estudios permiten investigar mejor una asociación del consumo del cannabis de forma genética con la periodontitis. De los estudios hallados al respecto, se ha encontrado una baja evidencia de la responsabilidad genética por consumir cannabis que influye en la periodontitis. A partir de ello, una de las recomendaciones es replicar los hallazgos mediante estudios más extensos acerca de la asociación de todo el genoma y que tengan mayores exposiciones del cannabis fenotipado con mayor precisión ^25^.

Es importante considerar toda esta información del cannabis y con qué frecuencia es consumido por los pacientes para saber cómo actuar frente y qué plan de tratamiento, prevención y promoción de la salud seguir. Por ejemplo, antes de comenzar el tratamiento de ortodoncia, se debe conocer esos detalles, ya que durante este tratamiento se producen remodelaciones óseas y movimiento dental, y los fumadores de cannabis pueden tener movimientos dentales ineficientes y la duración de la ortodoncia sería mayor. Asimismo, se pueden generar reabsorciones óseas, perdida óseas, inflamación periodontal y mayor riesgo de desmineralización del tejido del esmalte ^49^.

### El papel de los profesionales dentales en el abordaje de pacientes fumadores de tabaco, derivados nicotínicos y cannabis

El cigarrillo y sus derivados, así como el cannabis, representan uno de los factores predisponentes frecuentes en la afectación del tejido periodontal y bucal, y generan también un riesgo de cáncer oral. La constitución de estas sustancias, como nitrosodietanolamina, nicotina, cannabinoides, entre otras, constituye una fuente de daño químico a la cavidad bucal y sus tejidos ^50^.

Por otra parte, tenemos el uso de tabaco sin humo, que involucra el uso de tabaco masticable y rapé, así como la masticación de paan, gutka o aspirar hojas sueltas. Incluso, el rapé es usado en edulcorantes y aromatizantes ^42^. Entre las consecuencias de esta droga tenemos las recesiones gingivales, las bolsas periodontales, el sangrado gingival, así como la posible aparición de alteraciones en el gusto, manchas en las piezas dentarias, halitosis, úlceras, entre otros ^46^.

Los efectos adversos de dichas sustancias pueden generar la aparición de lesiones precancerosas y cancerosas, así como movilidad dentaria y pérdida de piezas dentarias por la periodontitis generada. Por ello, diversos estudios han demostrado la asociación de estas drogas con la enfermedad periodontal. Entre los estudios transversales realizados sobre el tema, se demostró que la asociación entre estos productos puede causar lesiones precancerosas y cancerosas, como lesiones bucales de menor gravedad. Sin embargo, se evidencia una asociación directa y significativa del tiempo de uso de estos productos con lesiones bucales como periodontitis, recesiones gingivales, pérdida dentaria, entre otras ^41^. Otro punto es que la exposición a estos productos con nicotina altera la microbiota oral, lo que genera la proliferación de patógenos que causan enfermedades bucales. Y esto se debe a un cambio de bacterias aerobias comensales a anaerobias ^51^.

Por otro lado, ha aumentado el uso de cigarrillos electrónicos en los últimos años, ya que, a diferencia de los cigarrillos tradicionales, estos administran la nicotina mediante líquidos de vapeo en aerosoles. A causa del incremento reciente en su uso, existe poca evidencia de las consecuencias de los cigarrillos electrónicos en la cavidad bucal. En los estudios encontrados, se revela que el uso continuo de cigarrillos electrónicos también incrementa la disbiosis microbiana que conduce a la enfermedad periodontal. Se evidencia una asociación significativa con inflamaciones gingivales y alteración de la microbiota oral a nivel subgingival y en la saliva ^2^.

Entre otros estudios, aquellos que fueron realizados en colegios, se halló que la edad promedio para empezar a fumar es a partir de los 14 años, y que más de la mitad consumían de 1 a 10 cigarrillos al día. Así, se evidencia una asociación directa entre la edad y la intensidad del uso del tabaco, además de una baja salud periodontal adecuada también en poblaciones jóvenes. Por tanto, se debe hacer hincapié en los efectos negativos del consumo de tabaco sobre la salud periodontal, a fin de ejecutar más medidas de prevención del tabaquismo ^3^.

Por otro lado, los pacientes fumadores suelen presentar problemas tanto en su salud general como la dental, por lo que hay evidencia contundente de la asociación entre estas drogas con enfermedades bucodentales ^52^. Debido a este motivo, los odontólogos deben promover y asesorar a sus pacientes acerca de su salud dental y que ellos puedan conocer los efectos adversos que trae el tabaquismo, para así aminorar el consumo de estas drogas ^(53, 54)^. Ante ello, el profesional también debe estar en constante capacitación, ya que con el paso del tiempo se obtiene más información sobre el tratamiento farmacológico y psicológico para la prevención de productos nicotínicos, tabaco y cannabis relacionado con el campo dental ^55^. Y resalta que el tratamiento puede variar y depende también de la conducta del paciente ^56^.

Muchos estudios resaltan el efecto del tabaquismo en el periodonto y demás tejidos dentales, por lo que es uno de los factores contribuyentes en la periodontitis y la pérdida de piezas dentarias, incluso al dejar de fumar ^57^^-^^59^. También suelen visualizarse altos niveles creviculares en este tipos de pacientes, como la proteína 1 del grupo de alta movilidad (HMGB-1) ^60^, por lo que es necesario la asesoría del profesional para aminorar posibles riesgos en la salud bucal y aplicar ciertos tratamientos que han sido evidenciados como efectivos, entre ellos la administración subgingival de clorhidrato de minociclina para el desbridamiento mecánico no quirúrgico ^61^. Esto indica que el dejar de fumar reduciría el riesgo de pérdidas dentarias y otras complicaciones ^62^.

Se puede observar que, en la malla curricular y formación de los odontólogos, no se contempla este aspecto y no se aplican programas de promoción y prevención de la salud dental dirigidos a pacientes fumadores en las consultas ^63^^,^^64^. El odontólogo, al poseer los recursos necesarios para una intervención interdisciplinaria con otros profesionales de la salud en pacientes fumadores, podría influir en ellos durante la consulta dental, lo que generaría resultados positivos y eficaces. El uso de técnicas y tratamientos adecuados por parte de los dentistas, no solo en el aspecto dental, genera una atención más integral en este tipo de pacientes, por lo que ayuda a prevenir complicaciones y futuras enfermedades en la salud dental y general ^65^. 

El odontólogo cumple un papel importante también en el diagnóstico precoz de posibles enfermedades y en su tratamiento. Con el paso del tiempo, ha obtenido un rol reconocido en el equipo de profesionales de la salud por su ayuda continua y prevención de enfermedades. En el caso específico de la prevención y cese del consumo de tabaco y otras drogas, lleva a cabo un importante trabajo multidisciplinario con otros profesionales de la salud ^52^^,^^66^. Por tanto, el dentista tiene la responsabilidad de intervenir como parte del tratamiento dental y hacer uso de sus conocimientos sobre distintas estrategias durante las intervenciones de los pacientes fumadores, para brindarles una mejor calidad de vida, así como un servicio completo y eficaz ^67^.

## LIMITACIONES

En cuanto a las limitaciones en el presente estudio, no existe suficiente evidencia científica respecto de la relación entre el cannabis y la enfermedad periodontal. Asimismo, tras una exhaustiva búsqueda bibliográfica en distintas bases de datos, no se garantiza que hayan sido considerados todos los estudios; esto debido a que la presente revisión solo consideró aquellos estudios con una antigüedad de 11 años atrás hasta la actualidad, por lo que aquellas investigaciones con una mayor antigüedad no fueron consideradas. 

## CONCLUSIÓN

La presente revisión concluye que, de acuerdo con la evidencia recolectada, existe una asociación entre la enfermedad periodontal y el consumo de cigarrillos, derivados nicotínicos y cannabis. Estas drogas constituyen un factor predisponente y agravante de la enfermedad periodontal, que genera la aparición de bolsas periodontales, el aumento del cálculo gingival, entre otras características que afectan la salud oral. Sin embargo, se sugiere a futuras investigaciones el evaluar y poner más énfasis en la asociación del cannabis con la enfermedad periodontal, ya que la evidencia existente es escasa a diferencia de la que se tiene sobre otras drogas relacionadas con la enfermedad periodontal.

## Financiamiento:

nvestigación autofinanciada
